# Epigenetic Regulation of Dpp6 Expression by Dnmt3b and Its Novel Role in the Inhibition of RA Induced Neuronal Differentiation of P19 Cells

**DOI:** 10.1371/journal.pone.0055826

**Published:** 2013-02-07

**Authors:** Muhammad Abid Sheikh, Yousra Saeed Malik, Huali Yu, Mingming Lai, Xingzhi Wang, Xiaojuan Zhu

**Affiliations:** Institute of Genetics and Cytology, Northeast Normal University, Changchun, China; Muséum National d’Histoire Naturelle, France

## Abstract

DNA methylation is an important mechanism of gene silencing in mammals catalyzed by a group of DNA methyltransferases including Dnmt1, Dnmt3a, and Dnmt3b which are required for the establishment of genomic methylation patterns during development and differentiation. In this report, we studied the role of DNA methyltransferases during retinoic acid induced neuronal differentiation of P19 cells. We observed an increase in the mRNA and protein level of Dnmt3b, whereas the expression of Dnmt1 and Dnmt3a was decreased after RA treatment of P19 cells which indicated that Dnmt3b is more important during neuronal differentiation of P19 cells. Dnmt3b enriched chromatin library from RA treated P19 cells identified dipeptidyl peptidase 6 (*Dpp6*) gene as a novel target of Dnmt3b. Further, quantitative ChIP analysis showed that the amount of Dnmt3b recruited on *Dpp6* promoter was equal in both RA treated as well as untreated p19 cells. Bisulfite genomic sequencing, COBRA, and methylation specific PCR analysis revealed that *Dpp6* promoter was heavily methylated in both RA treated and untreated P19 cells. Dnmt3b was responsible for transcriptional silencing of *Dpp6* gene as depletion of Dnmt3b resulted in increased mRNA and protein expression of Dpp6. Consequently, the average methylation of *Dpp6* gene promoter was reduced to half in Dnmt3b knockdown cells. In the absence of Dnmt3b, Dnmt3a was associated with *Dpp6* gene promoter and regulated its expression and methylation in P19 cells. RA induced neuronal differentiation was inhibited upon ectopic expression of Dpp6 in P19 cells. Taken together, the present study described epigenetic silencing of Dpp6 expression by DNA methylation and established that its ectopic expression can act as negative signal during RA induced neuronal differentiation of P19 cells.

## Introduction

Epigenetic organization of gene expression involves DNA methylation, histone modifications, chromatin remodeling, and RNA interference. These mechanisms control many important cellular functions, including cell proliferation, differentiation, and development [1]. DNA methylation represents covalent modification of the cytosine residues at the CpG islands which are found in the proximal promoter regions of almost 50% of mammalian genes. Silencing of gene expression by DNA methylation is carried out by either lack of transcription factor binding to methylated DNA [2] or recruitment of methyl-CpG-binding domain (MBD) proteins which in turn bind with histone deacetylases (HDACs) to form a large repressor complex at the promoter region [3]. DNA methylation is catalyzed by DNA methyltransferases (Dnmts) that consist of a family of enzymes including Dnmt1, Dnmt3a, and Dnmt3b [4], [5]. Dnmt1 is a major maintenance methylation enzyme as it acts on hemimethylated DNA and copies the methylation pattern during DNA replication [6]. Dnmt3a and Dnmt3b are involved in the establishment of new methylation patterns during development, and hence they are the *de novo* methyltransferase enzymes [7]. Targeted mutation of Dnmts results in genomic demethylation and embryonic lethality in mice, indicating their essential role in embryo development [8], [9]. Dnmt1 and Dnmt3b null mice die during gestation period, whereas Dnmt3a null mice die shortly after birth [10].

DNA methylation is a reversible process and subjected to dynamic regulation during development. Adult methylation pattern of a particular cell is established through waves of demethylation and *de novo* methylation to carry out cell and tissue specific gene expression during development [11], [12]. In order to study the role of DNA methylation during neuronal differentiation, we selected P19 cells which are pluripotent stem cells that can be either maintained in the proliferating stage or efficiently induced to neuronal morphology by using retinoic acid (RA). P19 cells have been widely used as a model to understand the different aspects of differentiation [13], [14]. In the present study, we observed selective up-regulation of Dnmt3b and identified *Dpp6* gene as its novel target in P19 cells.

Dpp6 is a member of dipeptidyl peptidase IV family of proteins which regulate diverse biological functions including cell differentiation, apoptosis, proliferation, and carcinogenesis [15], [16]. Dpp6 is an integral membrane glycoprotein which consists of a large extracellular C-terminal domain, a membrane spanning region, and a short N-terminal domain [17], [18]. It has been suggested that Dpp6 is involved in the modulation of A-type potassium channels in neurons and thus play an important role in synaptic plasticity [19], [20]. Dpp6 is also involved in the maintenance of cell-specific phenotype and its deregulation can result in carcinogenesis. Hypomethylation and increased expression of *Dpp6* gene is found in colon cancer [21]. In contrast, hypermethylation and reduced expression of Dpp6 is observed in melanoma [22] and acute myeloid leukemia (AML) patients [23]. Differential expression of Dpp6 is found during embryogenesis and in adult tissues [24], [25]. Moreover, Dpp6 is expressed in different regions of the adult mouse brain and is regulated in a temporal and spatial manner during CNS development [26], [27], [28]. These studies clearly indicate that Dpp6 must be tightly regulated during neoplastic transformation and development. The present study deals with the epigenetic silencing of Dpp6 expression by DNA methylation and established that its ectopic expression can negatively regulate RA induced neuronal differentiation of P19 cells.

## Materials and Methods

### Antibodies

The following antibodies were used for ChIP analysis, immunostaining, and western blot analysis. Dnmt1 (IMG-261A), Dnmt3a (IMG-268A), Dnmt3b (IMG-184A) were purchased from Imgenex Corp. Antibodies against MAP2 (M 9942) and β-actin (A5441) were purchased from Sigma Aldrich. Anti-β III tubulin (TU-20, ab18207) and anti-Dpp6 (ab41809) were purchased from Abcam.

### Cell Culture and Transfections

All chemicals for cell culture were purchased from GIBCO unless otherwise stated. For lentivirus production, HEK 293FT cells were grown using DMEM and 10% FBS. Undifferentiated P19 cells (ATCC) were routinely cultured in DMEM/F12 medium supplemented with 10% fetal bovine serum (FBS). Differentiation of P19 cells into neuronal cells was performed as described previously [29]. P19 cells (5×10^3^ per cm^2^) were seeded onto gelatin-coated tissue culture dishes under full serum conditions for 24 h. After that cells were cultured under serum free conditions in DMEM/F12 medium with Insulin, Transferrin, Selenium (ITS) supplement. Cells were treated with 0.5 µM all-trans RA (Sigma) for initial 2 days of serum free conditions. After that, RA is removed from the medium and the cells were further cultured under serum free conditions.

Stable transfections of P19 cells were performed using Fugene6 reagent (Roche) following manufacturer instructions. Briefly, 3×10^5^ P19 cells were plated per 60 mm dish one day before transfection. Next day, the cells were transfected by mixing plasmid DNA (pCMV and pCMV-Dpp6, Origene) with Fugene6 in serum free media. The mixture was incubated at room temperature for 15 min and added dropwise to cells. For stable transfections, G418 (1 mg/ml) was added to culture medium 2 days after transfection to select for transfected cells.

### Western Blot Analysis

Western blot analysis was performed as described previously [30]. Briefly, total cell protein was extracted by direct lysis of cells attached to tissue culture plates using RIPA lysis buffer supplemented with protease inhibitor cocktail (Roche). The protein samples were resolved by SDS-PAGE, transferred to PVDF membrane and subjected to immunoblot analysis with primary antibody dilutions overnight at 4°C. Next day, the membranes were washed with PBST and incubated with appropriate horseradish peroxidase-conjugated secondary antibodies for 1 h at room temperature. Protein bands were detected using ECL solution (Amersham) and exposure to X-Ray films.

### Immunocytochemistry

Cells were grown on glass coverslips, fixed in 4% paraformaldehyde, and permeabilized with 0.3% Triton X-100. The cells were then blocked for nonspecific binding by using 2% BSA in PBS for 1 h at room temperature and incubated with primary antibodies. The cells were washed with PBS and incubated with appropriate Alexa Flour® conjugated secondary antibodies (Invitrogen) at 1∶1000 dilution for 1 h at room temperature. Finally, the samples were washed with PBS, counterstained with DAPI (Invitrogen) to stain nuclei, mounted on glass slides and visualized by using confocal laser scanning microscope (OlympusFV1000).

### Real-time Quantitative RT-PCR Analysis

Total RNA from P19 cells was isolated using Trizol reagent (Biobasic) and 5µg was used to synthesize cDNA using M-MLV reverse transcriptase following the manufacturer’s protocol (Invitrogen). Real time PCR reactions were performed using SYBR® Green Realtime Master Mix and ABI PRISM® 7700 (Applied Biosystems). Primers are as follows: Dnmt1∶5′ –GAGAAGGACAAAGCACCCACG-3′ and 5′ –CACAGACACCACAGCGGCG-3′. Dnmt3a: 5′–GAAGCGGAGTGAACCCCAAC-3′ and 5′-CCTTGGTCACACAGCAGCC-3′. Dnmt3b: 5′-GCCAGCCTCACGACAGGAAAC-3′ and 5′-GACTGGGGGTGAGGGAGCATC-3′. Dpp6-RT: 5′ –GGGGTCTAGACATGTCCCCTCCCT-3′ and 5′–GCCAAGGGTCGGAGTGATCGC-3′. GAPDH: 5′-CAGTGGCAAAGTGGAGATTG-3′ and 5′-AATTTGCCGTGAGTGGAGTC-3′. Relative mRNA expression was calculated by using 2^−ΔΔCt^ method and normalized to that of GAPDH.

### Chromatin Immunoprecipitation (ChIP) Analysis

ChIP assays were carried out using a kit from Millipore according to manufacturer’s instructions. The cells were cross-linked with 1% formaldehyde, lysed, and sonicated (Vibra Cell) at different conditions to optimize the shearing of genomic DNA with an average size of ∼500 bp. The sheared samples were diluted 10-fold in ChIP dilution buffer and precleared using 75 µl of salmon sperm DNA/protein G agarose beads for 1 h. 1% of supernatant was saved as an input control. Five microgram of Dnmt antibodies and 1 µg of normal mouse IgG (provided in the kit) were added to diluted chromatin and incubated at 4°C overnight with rotation. Immune complexes were collected, washed, eluted, and the cross-links were reversed by the addition of 0.2 M NaCl and incubation at 65°C overnight. The proteins and RNA was removed by proteinase K and RNase A treatment and DNA was purified by using spin columns. The eluted DNA was either used as a template for quantitative PCR analysis or treated with T4 DNA polymerase (Takara) to make it blunt-ended. For TA cloning, the blunt-ended DNA was treated with *Taq* DNA polymerase (Takara) to add a 3′ A and ligated into pGEM-T vector (Promega). The ligated vectors were transformed into chemically competent JM109 bacteria (Takara) and white bacterial colonies were analyzed directly by colony PCR for presence or absence of an insert. Positive clones were then sequenced by using T7 forward primer and analyzed using BLAST (NCBI). Quantitative real time PCR analysis was performed using ChIP DNA and the data was normalized to that of input DNA. The primers used for *Dpp6* promoter were 5′-CTCCACTTTCTCGCCTTCACC-3′ and 5′-GCAGTCGGTGCGTATCCCAG-3′. The value of IgG (negative control) was set as 1, and the results were presented as relative fold enrichment of IgG.

### Bisulfite Genomic Sequencing, COBRA, and MSP

P19 cells (1×10^4^) were directly subjected for bisulfite conversion by using the EZ DNA methylation-direct Kit (ZYMO Research) following manufacturer’s instructions. DPP-6 CpG island (CGI) was amplified by using unbiased primers 5′-TTGGAGGGTTTTTTAAGGAGG-3′ and 5′-TCCAAATCTATCCAAACCAAAAATA-3′. For bisulfite genomic sequence analysis, the PCR product was cloned into pGEM-T vector and 15 individual clones from each group were sequenced and analyzed quantitatively by using BISMA online software [31]. For COBRA analysis, the same PCR product was digested with TaqI and BstUI (Both from NEB) to determine the methylation status. Methylation specific PCR was performed by using bisulfite converted DNA and primers 5′-TACGTATCGATTGTATTTGTAGTCG-3′ and 5′-GCTCTACCCAAAACTACCTCG-3′ for methylated DNA and primers 5′-GGGATATGTATTGATTGTATTTGTAGTTG-3′ and 5′-ACACTCTACCCAAAACTACCTCACT-3′ for unmethylated DNA. The PCR products were analysed by using agarose gels to distinguish between methylated and unmethylated DNA.

### Lentiviral shRNA Vector Production and Infection

shRNA oligos were annealed and cloned into the HpaI and XhoI digested pLL3.7 vector which contained a GFP marker to monitor virus production and infection. The following sequences were used to generate shRNA for Dnmt3a: 5′-GGGACAAGAATGCTACCAAAG-3′; Dnmt3b: 5′-GCGGGTATGAGGAGTGCATTA-3′; and a negative control shRNA: 5′-GGTGGCCGTAGATATAGAGGT-3′. Lentivirus particles were generated by co-transfecting shRNA containing pLL3.7 along with packaging plasmids (psPAX2 and pMD2G) in 293FT cells by using lipofectamine 2000 reagent (Invitrogen). The viral supernatant was collected 48 and 72 hours post-transfection, clarified by low speed centrifugation, and concentrated using ultracentrifugation at 25,000 rpm for 90 min at 4°C. Viral pellets were resuspended in sterile PBS at 1/500 of the original volume and titers were determined using FACS analysis. P19 cells were infected twice with lentiviral particles (MOI = 5) in the presence of 8 µg/ml polybrene.

### BrdU Incorporation Assay

P19 cells, which were induced to differentiate for 4 days following RA treatment, were trypsinized and 4×10^5^ cells were grown on glass coverslips overnight. BrdU (Sigma Aldrich) was added to media at final concentration of 50 µM and cells were incubated for 4 hours. Cells were then fixed, denatured and immunostained with anti-BrdU antibody (Abcam).

### Apoptosis Measurement by Annexin V Staining

RA treated P19 cells were harvested and Annexin V-FITC kit (KeyGen Biotech, china) was used to label apoptotic cells following manufacturer recommendations. 1×10^5^ Cells were suspended in binding buffer and then labeled with annexin V-FITC and propidium iodide for 10 min in dark. Following incubation, cells were analysed by flow cytometry.

### Statistical Analysis

Data from at least three independent experiments is represented as standard error of the mean. Statistical analysis was performed using one-way ANOVA with appropriate *post hoc* tests with *p value* <0.05 to be considered as significant.

## Results

### Differentiation of P19 Cells using Serum-free and Monolayer Culture

**Figure 1:**
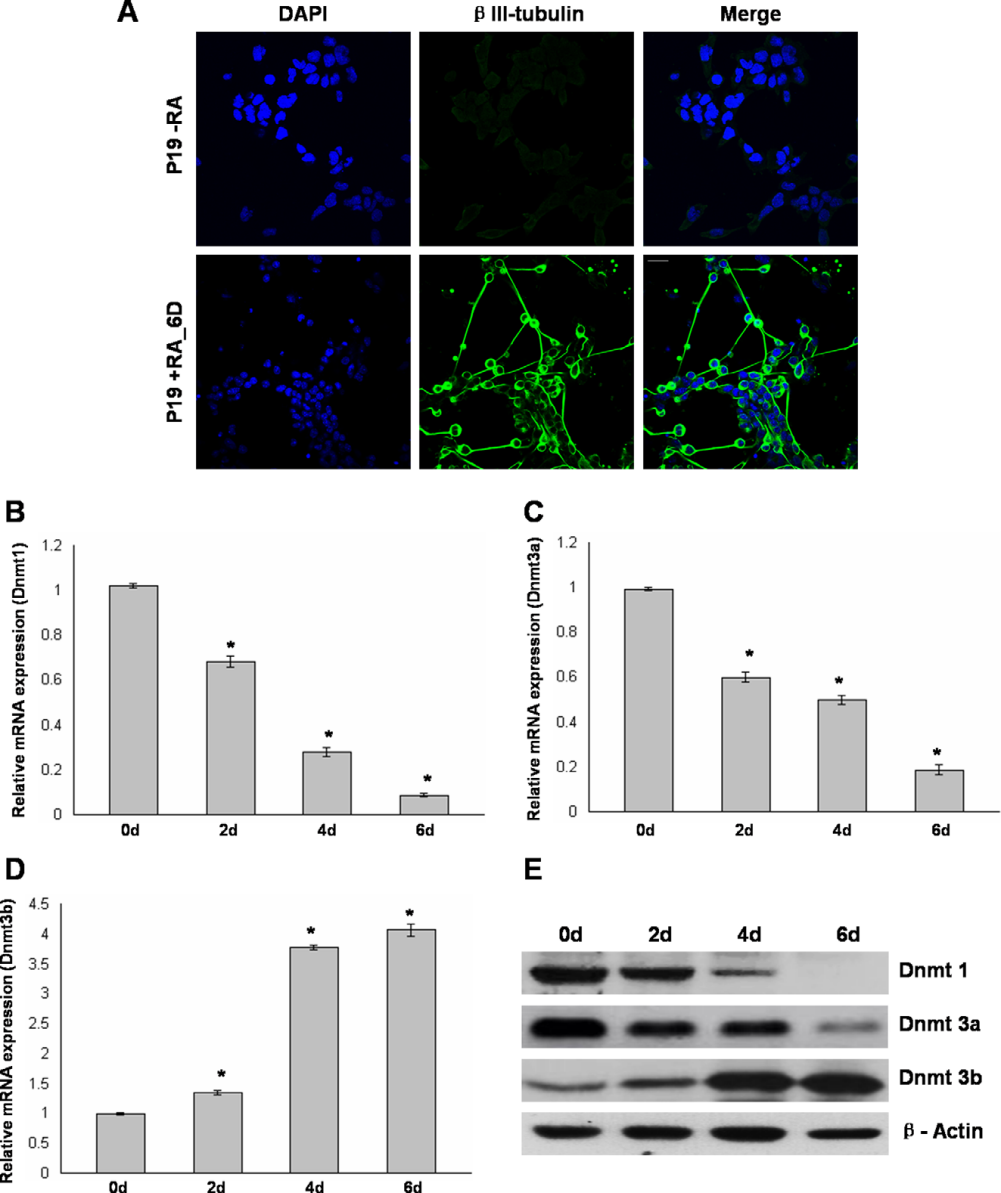
Differential expression of Dnmt1, Dnmt3a, and Dnmt3b during RA induced neuronal differentiation of P19 cells. A, P19 cells either left untreated (top panel) or RA treated for initial 2 days and further cultured for 4 days without RA (6 days, bottom panel) were immunostained with neuron specific β-III tubulin antibody and nuclei were stained using DAPI. B to D, Real time RT-PCR analysis of Dnmt1, Dnmt3a, and Dnmt3b during RA induced differentiation of P19 cells. Total RNA isolated from untreated P19 cells (D0) and RA treated P19 cells at different days (D2, D4, D6) was converted to cDNA and subjected to Real time quantitative PCR analysis using primers for Dnmt1, Dnmt3a, and Dnmt3b. GAPDH was used as an internal control while mRNA levels were relative to untreated P19 cells. Values represent mean of three independent experiments and error bars represent ± SEM. *P<0.05 versus untreated P19 cells. E, Western blot analysis revealed changes in the protein level of Dnmt1, Dnmt3a, and Dnmt3b at different days during P19 cell differentiation. Total cell protein from untreated P19 cells (D0) and RA treated P19 cells at different days (D2, D4, D6) was subjected to immunoblot analysis using Dnmt1, Dnmt3a, and Dnmt3b antibodies with β-Actin used as loading control. Scale bar: 20 µ m.

In this study, we used a simplified monolayer based serum-free strategy to induce P19 cells to neural lineage. P19 cells were exposed to 0.5 µM RA as monolayer for initial 2 days of culture under serum free conditions. After another 4 days of culture in serum-free media without RA, the cells were differentiated by rounding up and forming neurosphere-like colonies with expanding neurites. In order to confirm the neuronal morphology, the cells were stained for neuron specific βIII-tubulin (TU-20). RA induced P19 cells showed immunoreactivity against βIII-tubulin, indicating a neuronal phenotype. In contrast, undifferentiated P19 cells were βIII-tubulin negative (Fig. 1A).

### Differential Expression of DNA Methyltransferases During Differentiation of P19 Cells

As an initial step to study the role of DNA methyltransferases in RA induced neuronal differentiation of P19 cells, we examined the mRNA levels of Dnmt1, Dnmt3a, and Dnmt3b at different time periods during differentiation by real-time RT-PCR analysis. After 2 days of RA treatment, the level of Dnmt1 was reduced to ¾ of untreated P19 cells and it rapidly decreased at day 4 and day 6 of differentiation (Fig. 1B). A gradual decrease in the mRNA level of Dnmt3a was observed following RA treatment and it was subsequently maintained at the reduced level (Fig. 1C). Nearly 3 fold increase in the mRNA level of Dnmt3b was observed after 4 days of culture and it was further increased at day 6 of culture (Fig. 1D). Further, western blot analysis was used to analyze the protein levels of Dnmt enzymes during differentiation. The results correlated well with the RT-PCR analysis and we observed a selective increase in protein level of Dnmt3b. In addition, Dnmt1 and Dnmt3a were decreased in response to RA (Fig. 1E). These results suggested differential increase of dnmt3b expression during RA induced differentiation of P19 cells.

### Association of Dnmt3b with the Promoter of *Dpp6* Gene in P19 Cells

Increased expression of Dnmt3b following RA treatment led us to investigate its potential target genes in P19 derived neurons. For this purpose we utilized chromatin immunoprecipitation (ChIP) to generate a library of Dnmt3b-bound chromatin fragments. Sonication of formaldehyde fixed cells for ten 10 sec pulses (10 sec on, 10 sec off) resulted in chromatin fragments with an average size of approximately 500 bp for both RA treated and untreated P19 cells (Fig. S1). The sheared chromatin from RA treated P19 cells was immunoprecipitated with Dnmt3b antibody and the pulled-down DNA was modified for cloning into pGEM-T vector. Following transformation, initial analysis of 408 white bacterial colonies yielded 198 insert containing PCR products. Approximately 160 clones were successfully sequenced and identification of potential target genes was established by BLAST analysis. Based on their function, Dnmt3b target genes were classified into seven categories (Supporting information Table S1). Sequences that are not included in the analysis were either repetitive or did not produce a statistically significant homology (not listed).

**Figure 2:**
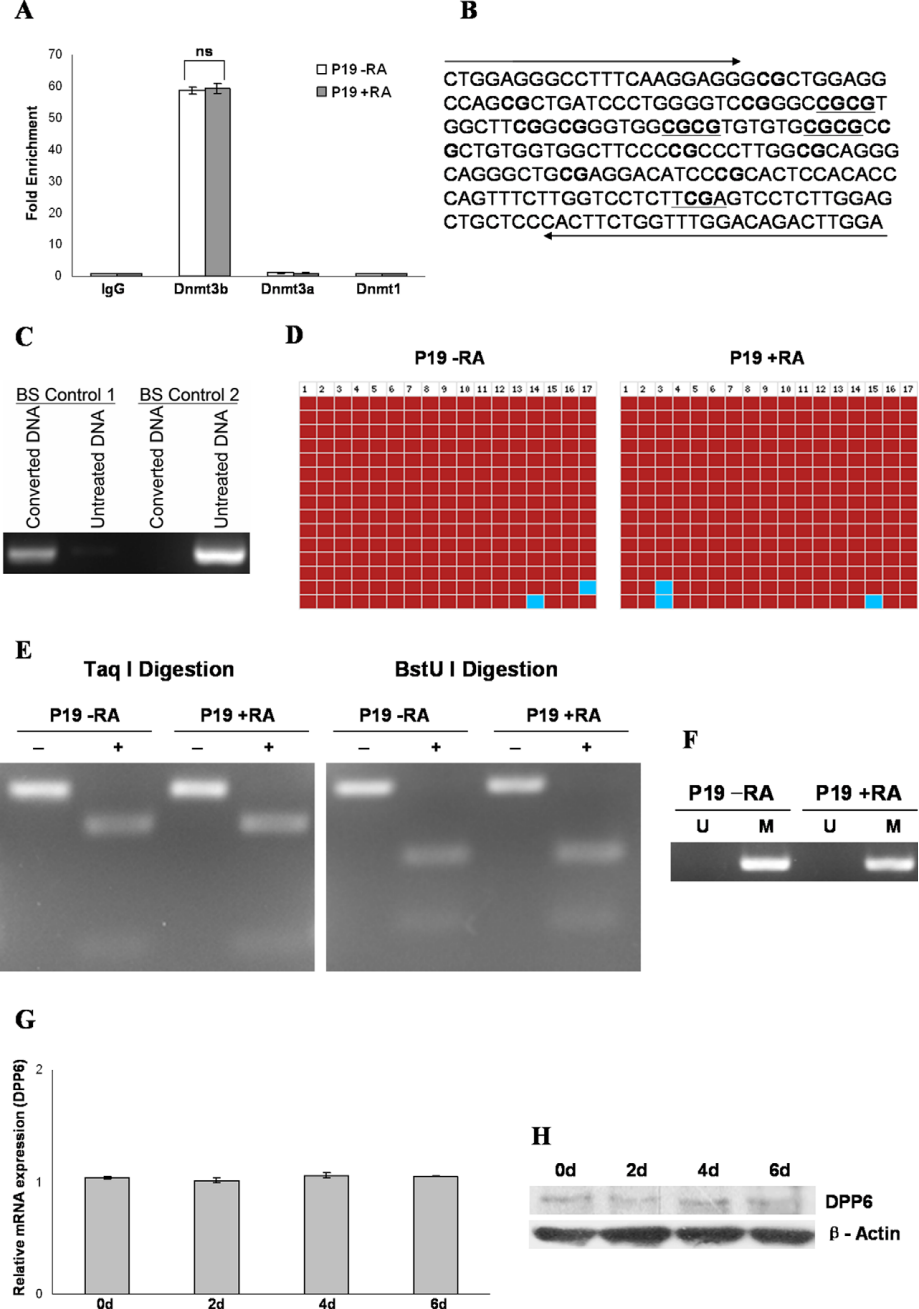
Association of Dnmt3b with the promoter of *Dpp6* gene, methylation pattern of *Dpp6* promoter CpG Island and its expression in P19 cells. A, Recruitment of Dnmt1, Dnmt3a, and Dnmt3b on *Dpp6* promoter in undifferentiated (P19 -RA) and differentiated P19 cells by RA treatment for initial 2 days and further culture for 4 days without RA by using quantitative ChIP analysis. Data was normalized to input fraction. The value of IgG (negative control) was set as 1, and the results were presented as relative fold enrichment of IgG. Values represent mean of three independent experiments and error bars represent ± SEM. ns = no significant difference between P19–RA and P19+RA. B, The sequence of 222 bp PCR product used for BGS, and COBRA. Arrows indicate the position of primers and 17 CpG dinucleotides included in this analysis are shown in bold. Restriction Sites for TaqI and BstUI enzymes are underlined. C, Bisulfite conversion control PCR by using primer sets that can distinguish between converted (Control 1) and unconverted DNA (Control 2). D, Bisulfite genomic sequence analysis of *Dpp6* promoter in undifferentiated (P19 -RA) and differentiated P19 cells by RA treatment for initial 2 days and further culture for 4 days without RA. 17 CpG sites were analyzed in 15 individual clones. The red and blue colored boxes showed methylated and unmethylated cytosines respectively. E, For COBRA analysis, the PCR products were digested with TaqI and BstUI followed by agarose gel electrophoresis to analyze methylation status at *Dpp6* promoter. F, Methylation Specific PCR was performed using bisulfite treated DNA and primers that can discriminate between unmethylated (U) and methylated (M) DNA. G, Quantitative real time RT-PCR analysis of Dpp6 in untreated P19 cells (D0) and RA treated P19 cells at different days (D2, D4, D6). Values represent mean of three independent experiments and error bars represent ± SEM. H, Western blot analysis showing protein expression of Dpp6 in RA untreated and RA treated P19 cells at different days.

We selected one of the target genes, *Dpp6*, for further study based on the fact that dipeptidyl peptidase proteins regulate diverse biological processes including adhesion, apoptosis, carcinogenesis, cell proliferation, and differentiation [15], [16]. In order to study the recruitment of Dnmt1, Dnmt3a, and Dnmt3b on *Dpp6* promoter, we again performed ChIP followed by quantitative PCR analysis. As compared to IgG (negative control), almost 60 fold enrichment of *Dpp6* promoter was observed with Dnmt3b pulled down DNA from both RA treated and untreated P19 cells. *Dpp6* promoter enrichment above background was not observed with either Dnmt1 or Dnmt3a pulled down DNA. Furthermore, the amount of Dmt3b associated with *Dpp6* promoter was equal in both RA treated as well as untreated p19 cells (Fig. 2A).

### Methylation Pattern of *Dpp6* Promoter CpG Island and its Expression in P19 Cells

Association of Dnmt3b with the promoter of *Dpp6* prompted us to examine the methylation pattern of *Dpp6* promoter CpG Island (Fig. 2B) in P19 cells. The first step for any type of methylation analysis is bisulfite treatment of genomic DNA which converts unmethylated cytosine to uracil, while 5-methylcytosine is resistant to this conversion. Following PCR, uracils and methylcytosines are recognized as thymines and cytosines, respectively. Complete bisulfite conversion of genomic DNA was verified by PCR with primers that can distinguish between unconverted and converted DNA (Fig. 2C). In order to determine the methylation status of individual CpG sites within *Dpp6* promoter, bisulfite genomic sequence (BGS) analysis was performed. For this purpose, a 222 bp PCR product was amplified and cloned. Analysis of 15 individual clones revealed that, 17 CpG dinucleotides were more than 98% methylated in both RA treated and untreated P19 cells (Fig. 2D). Next, the PCR product used for bisulfite genomic sequencing was digested with TaqI (TCGA) and BstUI (CGCG). After bisulfite modification, only methylated DNA could be digested by these enzymes as their recognition sequence contain cytosines which are replaced by Thymines in unmethylated DNA. Complete digestion of PCR products was observed which showed an almost 100% methylation at CpG island of *Dpp6* promoter in both types of P19 cells (Fig. 2E). We also performed methylation specific PCR (MSP) using bisulfite treated genomic DNA by designing primer pairs (U and M) that can directly distinguish between unmethylated and methylated DNA, respectively. MSP results corroborated well with the results of BGS and COBRA as PCR bands were only observed with the primer pair specific for methylated DNA (Fig. 2F). These results clearly demonstrated that the CpG Island present in the promoter region of *Dpp6* gene was heavily methylated in P19 cells which remained methylated after RA treatment. To investigate the correlation between methylation and expression, we studied the mRNA and protein level of Dpp6 in RA untreated and during different days of RA treated P19 cells. Real time PCR analysis (Fig. 2G) and western blot (Fig. 2H) showed that the expression of Dpp6 remained unchanged after RA treatment. Western blot also showed that Dpp6 is expressed at fairly reduced levels which correlated well with the methylation analysis.

### Down-regulation of Dnmt3b Resulted in Increased Expression and Decreased Methylation of *Dpp6* Gene

**Figure 3:**
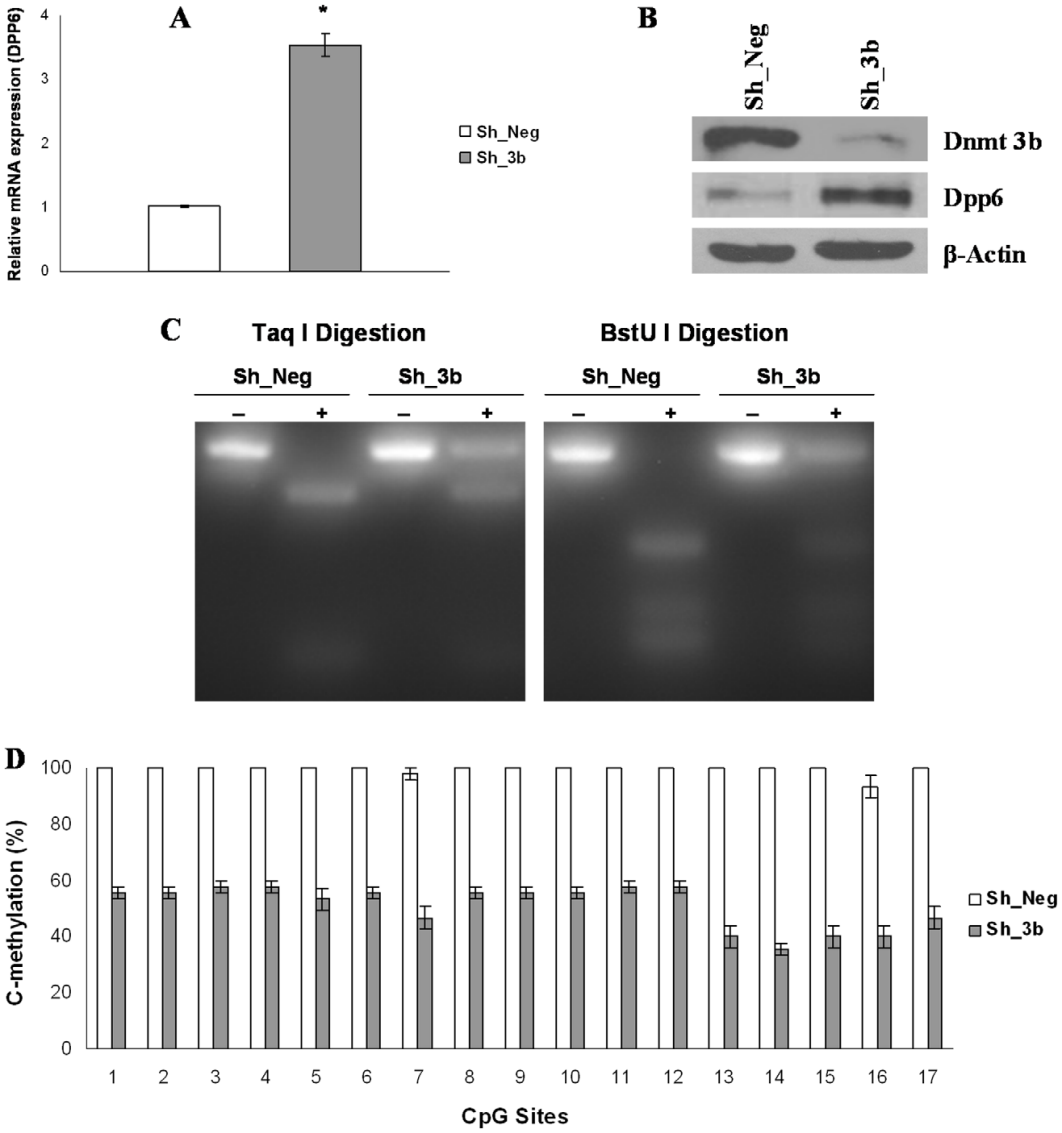
Depletion of Dnmt3b resulted in increased expression and decreased methylation of *Dpp6* gene in P19 cells. A, Real time RT-PCR analysis of Dpp6 in Dnmt3b depleted cells relative to negative control. Values represent mean of three independent experiments and error bars represent ± SEM. *P<0.05 versus negative control. B, Western blot analysis showing protein expression of Dnmt3b and Dpp6 in negative control and in Dnmt3b knockdown cells with β-Actin used as loading control. C, COBRA analysis of *Dpp6* promoter showing Taq I and BstU I digestion of *Dpp6* promoter in Dnmt3b depleted and negative control cells. Partial digestion is obvious in dnmt3b depleted cells as compared to control which is completely digested. D, Graph showing bisulfite genomic sequence analysis of *Dpp6* gene promoter in Dnmt3b depleted and negative control cells. Average % cytosine methylation analysis of 17 CpG sites in 15 different clones showed significant reduction of methylation in Dnmt3b depleted cells (P<0.05 versus negative control). Values represent mean of three independent experiments and error bars represent ± SEM.

The results presented in the previous sections documented that the promoter of *Dpp6* gene was methylated by Dnmt3b in P19 cells and their neuronal counterparts. For further elucidation, we used lentiviral shRNA to knockdown Dnmt3b and examined its effect on expression and methylation of *Dpp6* gene. Using optimum titers, >90% cells were GFP positive (Fig. S2A) and infection efficiency was quantified using flow cytometry (Fig. S2B). Western blot analysis showed significant depletion of Dnmt3b in cells expressing Dnmt3b shRNA as compared to control (Fig. 3B). Next, we examined the expression of *Dpp6* gene in Dnmt3b knockdown cells. Real time RT-PCR analysis showed that the Dpp6 mRNA was increased 3.5 fold in Dnmt3b shRNA expressing cells as compared to negative control (Fig. 3A). Western blot correlated well with the mRNA analysis and we observed increased expression of Dpp6 protein in Dnmt3b knockdown cells (Fig. 3B). These findings demonstrated that Dnmt3b negatively regulates Dpp6 expression and is involved in the silencing of *Dpp6* gene in P19 cells.

We also studied the effect of Dnmt3b knockdown on the methylation status of *Dpp6* gene promoter. COBRA analysis showed a significant decrease in the digestion of *Dpp6* gene promoter in Dnmt3b shRNA cells as compared to negative control which showed complete digestion with Taq I and BstU I restriction enzymes (Fig. 3C). In addition, bisulfite genomic sequencing revealed that the average methylation at 17 CpG sites in 15 different clones was reduced to 50% in Dnmt3b knockdown cells as compared to 99% in negative shRNA infected cells (Fig. 3D). Also, the effect of decreased methylation in Dnmt3b depleted cells was more pronounced at CpG sites 13–17 which are close to the transcription start site. Taken together, these results clearly demonstrated that Dnmt3b knockdown resulted in increased expression and decreased methylation of *Dpp6* gene in P19 cells.

### In the Absence of Dnmt3b, Dnmt3a Regulated the Expression and Methylation of *Dpp6* Gene in P19 Cells

**Figure 4:**
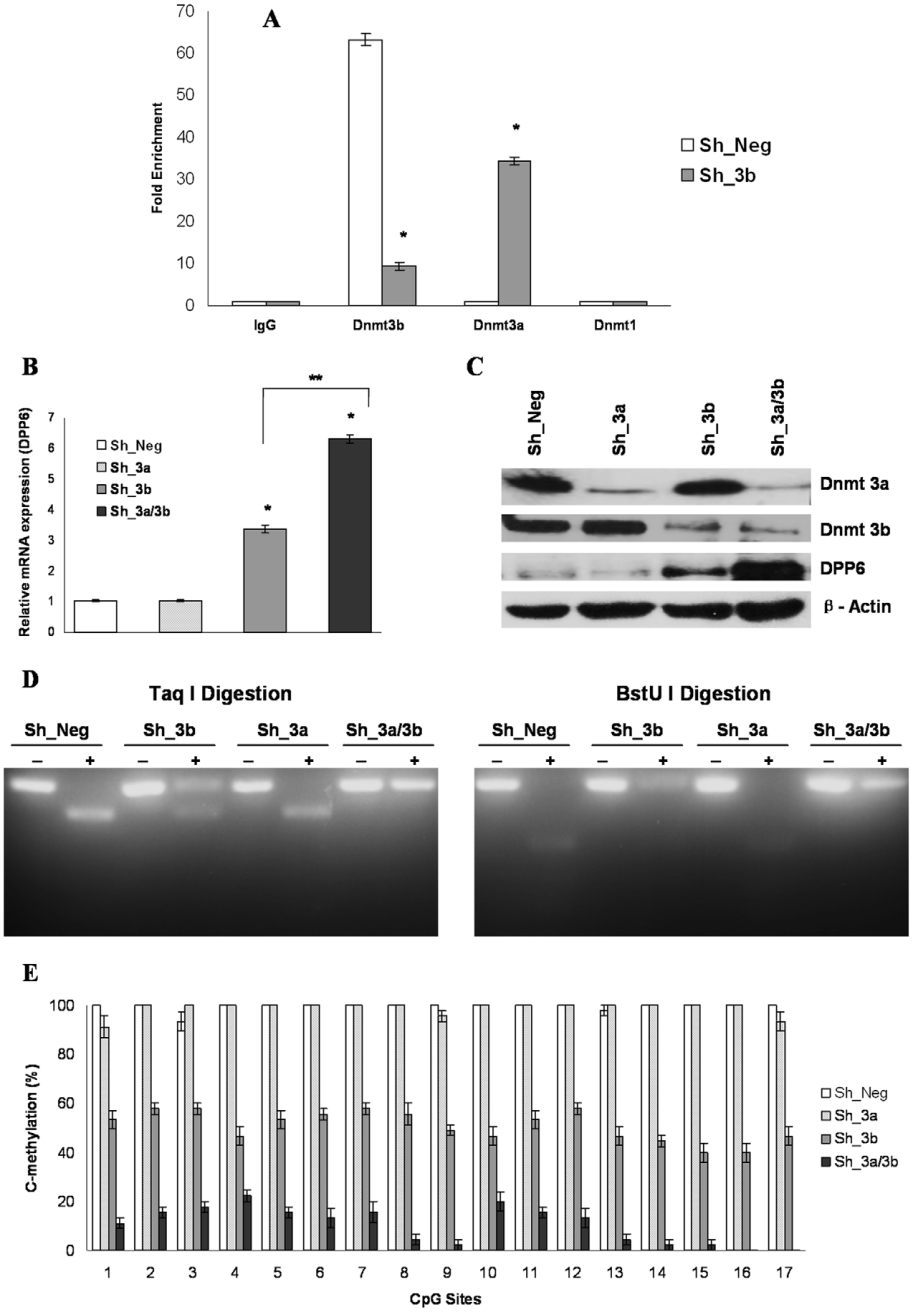
In the absence of Dnmt3b, Dnmt3a is associated with *Dpp6* gene promoter and regulates its expression and methylation in P19 cells. A, Quantitative ChIP assay was performed with Dnmt3b depleted and negative control cells using IgG and Dnmt antibodies followed by amplification of *Dpp6* promoter. Data was normalized to input fraction and the results were relative to that of IgG which was set 1. Values represent mean of three independent experiments and error bars represent ± SEM. *P<0.05 versus negative control. B, Real time RT-PCR analysis of Dpp6 mRNA in either single or double knockdown Dnmt3a and dnmt3b P19 cells relative to negative control. Values represent mean of three independent experiments and error bars represent ± SEM. *P<0.05 versus the negative control. **P<0.05 versus the single dnmt3b depleted cells. C, Western blot analysis showing specific depletion of Dnmt3a and Dnmt3b results in increased expression of Dpp6 protein in P19 cells. D, Taq I and BstU I digestion of *Dpp6* promoter in P19 cells either infected with single shRNA of Dnmt3a and Dnmt3b or in combination compared with negative control. The digestion of *Dpp6* promoter is almost abolished after depletion of both Dnmt3a and Dnmt3b as compared to single knockdown of Dnmt3b. E, Graph showing average % cytosine methylation at 17 CpG sites of *Dpp6* promoter in 15 different clones that were analyzed in either single Dnmt3a and Dnmt3b or double knockdown P19 cells. Statistically, no significant difference is observed between negative control and Dnmt3a depleted cells. However, single Dnmt3b and double knockdown of dnmt3a and Dnmt3b resulted in marked differences (P<0.05 versus negative control). Also, a significant difference is observed between single knockdown of Dnmt3b and depletion of both dnmt3a and Dnmt3b (P<0.05 versus Dnmt3b depleted cells). Values represent mean of three independent experiments and error bars represent ± SEM.

Since *Dpp6* gene promoter was still 50% methylated in the absence of Dnmt3b, it raised the question whether Dnmt1 and/or Dnmt3a were responsible for maintaining residual methylation of *Dpp6* gene. In order to test this hypothesis, we performed quantitative ChIP analysis using negative control and Dnmt3b depleted cells. Decreased abundance of Dnmt3b at *Dpp6* promoter was observed in knockdown cells as compared to control. In the absence of Dnmt3b, fold enrichment of Dnmt3a at *Dpp6* promoter increased significantly as compared to negative control which showed that only in the absence of Dnmt3b, Dnmt3a was associated with *Dpp6* promoter region (Fig. 4A). We did not find any enrichment of *Dpp6* promoter using Dnmt1 bound chromatin in both Dnmt3b knockdown and control cells. In order to prove above results, we used Dnmt3b and Dnmt3a depleted cells individually or in combination (Fig. 4C) and studied the effect on expression and methylation pattern of *Dpp6* gene. Real time RT-PCR analysis showed that as compared to negative control, more than 6 fold increase in the mRNA level of Dpp6 was observed in double knockdown cells which was also significantly higher than the expression observed in only Dnmt3b depleted cells (Fig. 4B). Knockdown of Dnmt3a alone did not influence the expression of Dpp6 as it was comparable to that of negative control. In addition, western blot also confirmed the results of mRNA analysis as highest Dpp6 protein expression was observed in Dnmt3a/Dnmt3b double knockdown cells (Fig. 4C). Concomitantly, both COBRA and bisulfite genomic sequencing showed that as compared to negative control (99.4% methylated), the average methylation of *Dpp6* promoter was reduced to 9.3% in double knockdown cells which is considerably lower than 50.8% methylation in single Dnmt3b depleted cells (Fig. 4D and E). As expected, Dnmt3a knockdown alone showed similar level of methylation to that of control. These results clearly demonstrated that only in the absence of Dnmt3b, Dnmt3a controls the expression and methylation of *Dpp6* gene in P19 cells.

### Ectopic Dpp6 Expression Resulted in Impaired Neuronal Differentiation of P19 Cells

**Figure 5:**
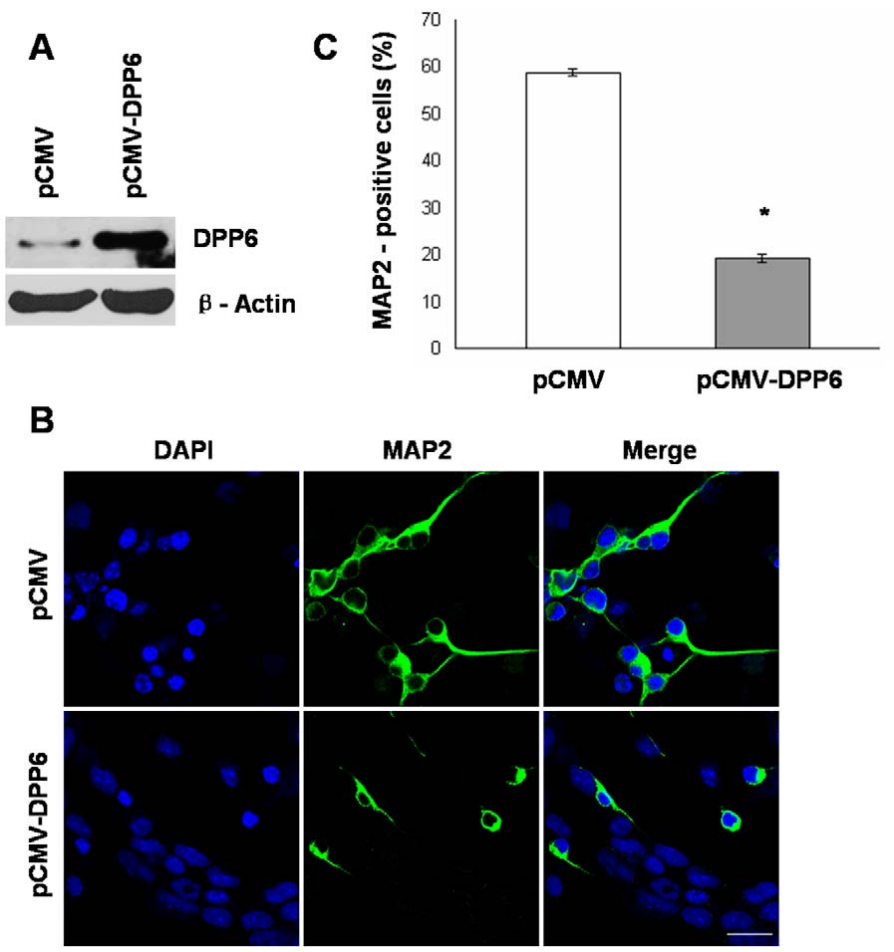
Ectopic expression of Dpp6 results in impaired neuronal differentiation of P19 cells. A, Western blot analysis showing expression of Dpp6 protein in stably transfected P19 cells either with empty vector (pCMV) or Dpp6 expression vector (pCMV-Dpp6). B, Stably transfected P19 cells with either pCMV or pCMV-Dpp6 were treated with RA initially for 2 days and further cultured for 4 days without RA treatment were immunostained with MAP-2 antibody and counterstained using DAPI. C, Graph showing % MAP-2 positive cells after 6 days differentiation of control and Dpp6 expressing P19 cells. At least 500 cells were counted from each group. Values represent mean of three independent experiments and error bars represent ± SEM. *P<0.05 versus the empty vector control. Scale bar: 20 µ m.

To explore the potential role of Dpp6 in RA induced neuronal differentiation, stable P19 cells expressing Dpp6 were generated. Western blot analysis confirmed the over-expression of Dpp6 in P19 cells transfected with pCMV-Dpp6 (Fig. 5A) which were further used in this study. First, P19 cells expressing high levels of Dpp6 and empty vector control cells were induced by RA treatment to examine neuronal differentiation by immunostaining of neuronal marker, MAP2. As illustrated in (Fig. 5B), the control showed high percentage of MAP2 positive cells, whereas the number of MAP2 positive cells was significantly reduced in P19 cells expressing high levels of Dpp6. Almost 60% cells were MAP2 positive in control as compared to only 20% in over-expressing P19 cells (Fig. 5C). These studies established a negative effect of Dpp6 expression on RA induced neuronal differentiation of P19 cells.

**Figure 6:**
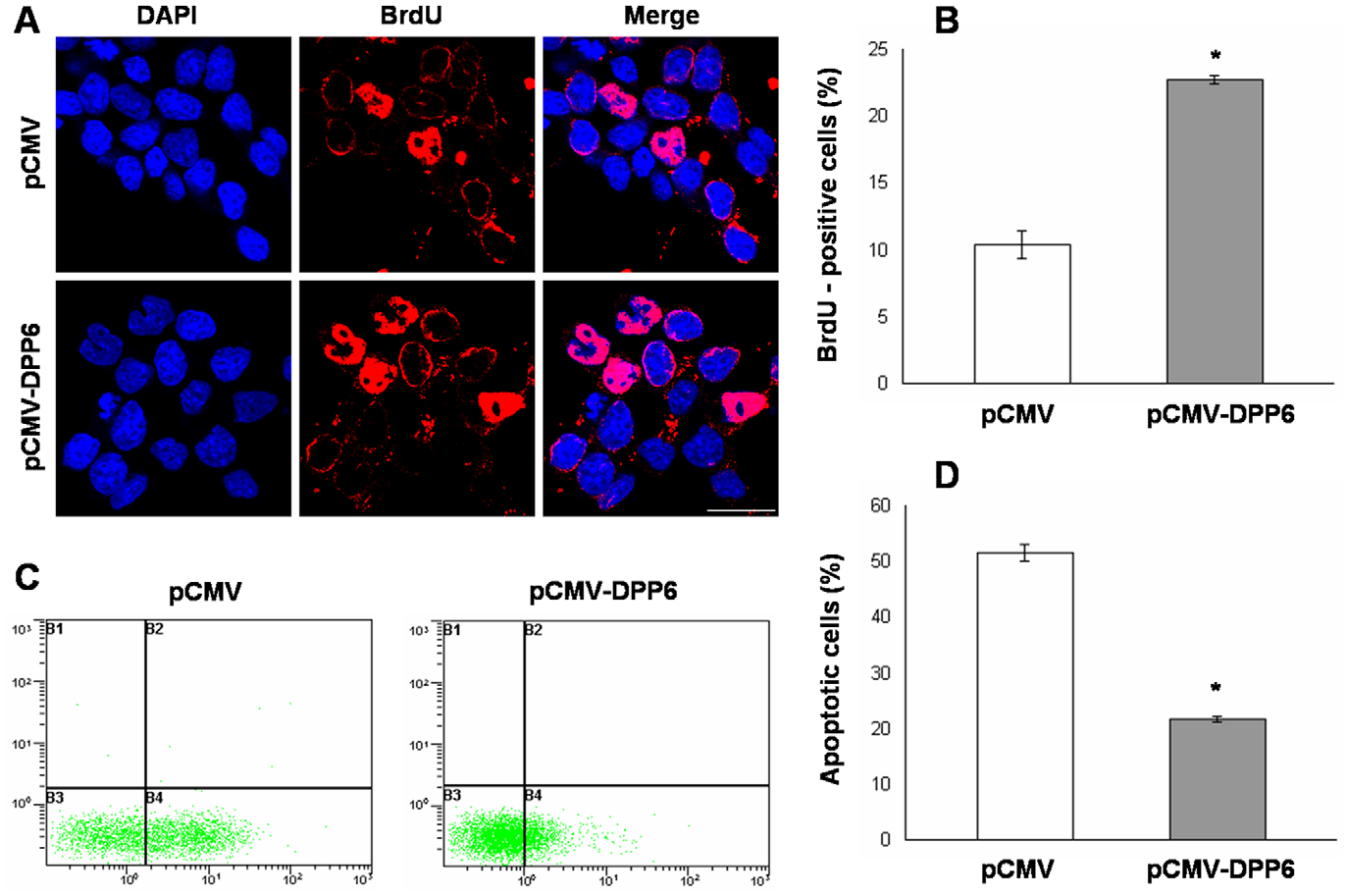
Dpp6 over-expression affects cell proliferation and apoptosis in RA induced P19 cells. A, Photograph showing BrdU incorporation in control (top) and Dpp6 over-expressing (bottom) P19 cells after 2 days of RA treatment and further culture for 2 days without RA. Cells were incubated with BrdU for 4 hrs, fixed and immunostained using antibody for BrdU while nuclei were detected using DAPI. B, Graph showing % of cells labeled with BrdU in control and Dpp6 expressing P19 cells after 4 days of differentiation. At least 500 cells were counted from each group. C&D, Measurement of apoptosis in control and Dpp6 expressing P19 cells after 2 days of RA treatment and further culture for 4 days without RA by labeling with Annexin V-FITC and propidium iodide. % apoptotic cells were quantified using flow cytometry analysis. Values represent mean of three independent experiments and error bars represent ± SEM. *P<0.05 versus the empty vector control. Scale bar: 20 µ m.

As neuronal differentiation is closely linked with cell proliferation and apoptosis, we also studied the effect of Dpp6 over-expression on these cellular processes. BrdU labeling was used to assess cell proliferation as BrdU is selectively incorporated into the DNA of S phase cells which are indicative of proliferating cells. The results showed that the number of BrdU positive cells was 23% in Dpp6 over-expressing cells as compared to control which showed only 10% cells as BrdU positive after RA induction (Fig. 6A and B). Finally, ectopic Dpp6 expression resulted in 22% of apoptotic cells as compared to 52% apoptosis in normally differentiating cells transfected with empty vector (Fig. 6C and D). Collectively, these results demonstrated that cells with Dpp6 over-expression were not successfully differentiated, showed high percentage of proliferating cells and reduced apoptosis as compared to normally differentiated P19 cells after RA treatment.

## Discussion

DNA methylation is an epigenetic phenomenon responsible for gene silencing at transcription level. De novo methylation pattern is established by Dnmt3a and Dnmt3b during embryo development that is then faithfully maintained in cell divisions. Aberrant DNA methylation can result in cancer progression or abnormal development. Therefore, DNA methylation must be tightly regulated during differentiation and development. In the present study, we explored the role of DNA methylation during neuronal differentiation of P19 cells. We observed a selective increase of Dnmt3b level upon RA treatment of P19 cells which is consistent with the higher level of Dnmt3b in the developing CNS during early neurogenesis [32], [33], suggesting its important role in neuronal differentiation. In addition, Dnmt3b is detected at high levels in mouse neuronal ectoderm at E7.5 and is predominantly expressed in the forebrain and eye at later stages of mouse embryonic development [10]. We also detected a decrease in the level of Dnmt1 and Dnmt3a upon RA induction of P19 cells which is in agreement with the down-regulation of these Dnmt enzymes during neural stem cell differentiation [34]. Dnmt1 is also decreased during epidermal differentiation [35], mouse myoblast differentiation [36], and during neuronal differentiation of Embryonic stem (ES) cells [37]. The loss of Dnmt1 and Dnmt3a expression could be balanced by the higher level of Dnmt3b upon RA induction. Dnmt3b could possibly substitute for Dnmt1 as the former can act on both unmethylated and hemimethylated DNA [38]. Dnmt3a and Dnmt3b have overlapping functions in mouse development and are also dynamically expressed in the CNS [33]. Therefore, it is assumable that Dnmt3b could also compensate for the decreased expression of Dnmt3a after RA treatment of P19 cells.

In the present study, we observed suppression of Dpp6 expression by Dnmt3b in P19 cells and studied its functional significance. Transcriptional silencing by Dnmt enzymes could be mediated by methylation dependent or independent manner. This is due to the fact that all Dnmt enzymes harbor an N-terminal domain, in addition to C terminal catalytic domain, which can recruit transcriptional repressors in a methylation independent manner [39], [40], [41]. For example, Dnmt3L which lacks a catalytic domain can still function as a negative regulator of transcription [42]. Here we observed that silencing of Dpp6 was due to catalytic activity of Dnmt3b as *Dpp6* gene promoter was heavily methylated in P19 cells. Depletion of Dnmt3b resulted in increased protein expression and decreased methylation of *Dpp6* gene promoter. Previous reports also showed regulation of Dpp6 expression by DNA methylation in some cancers [21], [22], [23]. In addition, global DNA methylation analysis identified *Dpp6* gene to be methylated in SH-SY5Y neuronal cells [43]. However, present study identified Dnmt3b responsible for methylation of *Dpp6* gene promoter and provided detailed mechanism of Dpp6 regulation lacking in earlier studies.

We also observed that in the absence of Dnmt3b, Dnmt3a could partly recruit to the promoter of *Dpp6* gene and regulated its expression and methylation status. This observation is quite interesting as both enzymes are previously known to have highly related biochemical and functional properties [44], [45]. For example, double knockout mice of Dnmt3a and Dnmt3b show genome wide hypomethylation; however, single knockout of either Dnmt does not result in a significant change of overall pattern of de novo methylation [10]. Furthermore, both enzymes are involved in the synergistic methylation of *Oct4* and *Nanog* genes to control cell differentiation [46]. These examples show that the target sequences for Dnmt3a and Dnmt3b are somewhat related and both enzymes have overlapping functions as discussed above.

Cell division and differentiation are two fundamental steps in development of an organism. In this context, proliferation and differentiation are basic incompatible cellular conditions and mitotic cells if do not reprogram after induction of neuronal differentiation often results in cell apoptosis [47]. In this study, we established that Dpp6 expression was silenced in P19 cells as well as during terminal differentiation of these cells. However, when expressed ectopically, Dpp6 can inhibit RA induced neuronal differentiation with significant reduction in neuronal marker MAP-2. In agreement with this result, Dpp6 over-expressing cells which did not commit to terminal differentiation showed high percentage of proliferating S phase cells and subsequent reduced apoptosis even after RA treatment as compared to normally differentiating cells. In this perspective, it is noteworthy that deregulated dipeptidyl peptidase proteins can cause proliferative disorders [16]. For example, expression of DppIV is increased during glioma [48] and lung cancer development [49]. Similarly, hypomethylation and subsequent increased expression of Dpp6 is observed during colon cancer progression [21].

The negative effect of ectopic Dpp6 expression on neuronal differentiation of P19 cells is also supported by the fact that expression of Dpp6 is not detected during early neurogenesis of mouse embryonic development when active neuronal differentiation is in progress [27], [49]. As discussed above, high expression of Dnmt3b during early neurogenesis [10], [32], [33] together with decreased expression of Dpp6 at similar stages of mouse embryonic development [27], [50] supports our study that Dpp6 is regulated by Dnmt3b mediated DNA methylation. In conclusion, the present study described the epigenetic silencing of Dpp6 expression by DNA methylation and showed that its ectopic expression can inhibit RA induced neuronal differentiation of P19 cells.

## Supporting Information

Figure S1 **P19 cells either untreated or RA treated for initial 2 days and further cultured for 4 days without RA were formaldehyde fixed, lysed, and sonicated for ten 10 sec pulses (10 sec on, 10 sec off) to yield DNA fragments with an average size of ∼500 bp.**
(DOC)

Figure S2 **A&B, P19 cells were infected with lentiviral particles that contain GFP as marker to monitor infection efficiency.** At optimal dose, more than 90% cells were GFP positive as determined by flow cytometry.
(DOC)

Table S1 **Dnmt3b target genes in P19 derived neurons.**
(DOC)

## References

[1] Wu H, Sun YE (2006) Epigenetic regulation of stem cell differentiation. Pediatr Res 59: 21R–25R. 16549544 10.1203/01.pdr.0000203565.76028.2a

[2] Watt F, Molloy PL (1988) Cytosine methylation prevents binding to DNA of a HeLa cell transcription factor required for optimal expression of the adenovirus major late promoter. Genes Dev 2: 1136–1143. 3192075 10.1101/gad.2.9.1136

[3] Fan G, Hutnick L (2005) Methyl-CpG binding proteins in the nervous system. Cell Res 15: 255–261. 15857580 10.1038/sj.cr.7290294

[4] Jurkowska RZ, Jurkowski TP, Jeltsch A (2011) Structure and function of mammalian DNA methyltransferases. Chembiochem 12: 206–222. 21243710 10.1002/cbic.201000195

[5] Lan J, Hua S, He X, Zhang Y (2010) DNA methyltransferases and methyl-binding proteins of mammals. Acta Biochim Biophys Sin (Shanghai) 42: 243–252. 20383462 10.1093/abbs/gmq015

[6] Hermann A, Goyal R, Jeltsch A (2004) The Dnmt1 DNA-(cytosine-C5)-methyltransferase methylates DNA processively with high preference for hemimethylated target sites. J Biol Chem 279: 48350–48359. 15339928 10.1074/jbc.M403427200

[7] Chedin F (2011) The DNMT3 family of mammalian de novo DNA methyltransferases. Prog Mol Biol Transl Sci 101: 255–285. 21507354 10.1016/B978-0-12-387685-0.00007-X

[8] Li E, Bestor TH, Jaenisch R (1992) Targeted mutation of the DNA methyltransferase gene results in embryonic lethality. Cell 69: 915–926. 1606615 10.1016/0092-8674(92)90611-f

[9] Lei H, Oh SP, Okano M, Juttermann R, Goss KA, et al (1996) De novo DNA cytosine methyltransferase activities in mouse embryonic stem cells. Development 122: 3195–3205. 8898232 10.1242/dev.122.10.3195

[10] Okano M, Bell DW, Haber DA, Li E (1999) DNA methyltransferases Dnmt3a and Dnmt3b are essential for de novo methylation and mammalian development. Cell 99: 247–257. 10555141 10.1016/s0092-8674(00)81656-6

[11] Boatright JH, Nickerson JM, Borst DE (2000) Site-specific DNA hypomethylation permits expression of the IRBP gene. Brain Res 887: 211–221. 11134609 10.1016/s0006-8993(00)02990-5

[12] Jaenisch R, Bird A (2003) Epigenetic regulation of gene expression: how the genome integrates intrinsic and environmental signals. Nat Genet 33 Suppl: 245–254 10.1038/ng1089 12610534

[13] Resende RR, Majumder P, Gomes KN, Britto LR, Ulrich H (2007) P19 embryonal carcinoma cells as in vitro model for studying purinergic receptor expression and modulation of N-methyl-D-aspartate-glutamate and acetylcholine receptors during neuronal differentiation. Neuroscience 146: 1169–1181. 17418494 10.1016/j.neuroscience.2007.02.041

[14] Ulrich H, Majumder P (2006) Neurotransmitter receptor expression and activity during neuronal differentiation of embryonal carcinoma and stem cells: from basic research towards clinical applications. Cell Prolif 39: 281–300. 16872363 10.1111/j.1365-2184.2006.00385.x PMC6496783

[15] Sedo A, Kraml J (1994) Dipeptidyl peptidase IV in cell proliferation and differentiation. Sb Lek 95: 285–288. 8867699

[16] Kotackova L, Balaziova E, Sedo A (2009) Expression pattern of dipeptidyl peptidase IV activity and/or structure homologues in cancer. Folia Biol (Praha) 55: 77–84. 19545486 10.14712/fb2009055030077

[17] Strop P, Bankovich AJ, Hansen KC, Garcia KC, Brunger AT (2004) Structure of a human A-type potassium channel interacting protein DPPX, a member of the dipeptidyl aminopeptidase family. J Mol Biol 343: 1055–1065. 15476821 10.1016/j.jmb.2004.09.003

[18] Qi SY, Riviere PJ, Trojnar J, Junien JL, Akinsanya KO (2003) Cloning and characterization of dipeptidyl peptidase 10, a new member of an emerging subgroup of serine proteases. Biochem J 373: 179–189. 12662155 10.1042/BJ20021914 PMC1223468

[19] Nadin BM, Pfaffinger PJ (2010) Dipeptidyl peptidase-like protein 6 is required for normal electrophysiological properties of cerebellar granule cells. J Neurosci 30: 8551–8565. 20573902 10.1523/JNEUROSCI.5489-09.2010 PMC2916862

[20] Sun W, Maffie JK, Lin L, Petralia RS, Rudy B, et al (2011) Dpp6 establishes the A-type K(+) current gradient critical for the regulation of dendritic excitability in CA1 hippocampal neurons. Neuron 71: 1102–1115. 21943606 10.1016/j.neuron.2011.08.008 PMC3184237

[21] Irizarry RA, Ladd-Acosta C, Wen B, Wu Z, Montano C, et al (2009) The human colon cancer methylome shows similar hypo- and hypermethylation at conserved tissue-specific CpG island shores. Nat Genet 41: 178–186. 19151715 10.1038/ng.298 PMC2729128

[22] Jaeger J, Koczan D, Thiesen HJ, Ibrahim SM, Gross G, et al (2007) Gene expression signatures for tumor progression, tumor subtype, and tumor thickness in laser-microdissected melanoma tissues. Clin Cancer Res 13: 806–815. 17289871 10.1158/1078-0432.CCR-06-1820

[23] Saied MH, Marzec J, Khalid S, Smith P, Down TA, et al (2012) Genome wide analysis of acute myeloid leukemia reveal leukemia specific methylome and subtype specific hypomethylation of repeats. PLoS One 7: e33213. 22479372 10.1371/journal.pone.0033213 PMC3315563

[24] Nadal MS, Amarillo Y, Vega-Saenz de Miera E, Rudy B (2006) Differential characterization of three alternative spliced isoforms of DPPX. Brain Res 1094: 1–12. 16764835 10.1016/j.brainres.2006.03.106

[25] Wada K, Yokotani N, Hunter C, Doi K, Wenthold RJ, et al (1992) Differential expression of two distinct forms of mRNA encoding members of a dipeptidyl aminopeptidase family. Proc Natl Acad Sci U S A 89: 197–201. 1729689 10.1073/pnas.89.1.197 PMC48203

[26] de Lecea L, Soriano E, Criado JR, Steffensen SC, Henriksen SJ, et al (1994) Transcripts encoding a neural membrane CD26 peptidase-like protein are stimulated by synaptic activity. Brain Res Mol Brain Res 25: 286–296. 7808228 10.1016/0169-328x(94)90164-3

[27] Hough RB, Lengeling A, Bedian V, Lo C, Bucan M (1998) Rump white inversion in the mouse disrupts dipeptidyl aminopeptidase-like protein 6 and causes dysregulation of Kit expression. Proc Natl Acad Sci U S A 95: 13800–13805. 9811881 10.1073/pnas.95.23.13800 PMC24902

[28] Clark BD, Kwon E, Maffie J, Jeong HY, Nadal M, et al (2008) Dpp6 Localization in Brain Supports Function as a Kv4 Channel Associated Protein. Front Mol Neurosci 1: 8. 18978958 10.3389/neuro.02.008.2008 PMC2576564

[29] Pachernik J, Bryja V, Esner M, Kubala L, Dvorak P, et al (2005) Neural differentiation of pluripotent mouse embryonal carcinoma cells by retinoic acid: inhibitory effect of serum. Physiol Res 54: 115–122. 15717849 10.33549/physiolres.930526

[30] Yu H, Wang N, Ju X, Yang Y, Sun D, et al (2012) PtdIns (3,4,5) P3 recruitment of Myo10 is essential for axon development. PLoS One 7: e36988. 22590642 10.1371/journal.pone.0036988 PMC3349655

[31] Rohde C, Zhang Y, Reinhardt R, Jeltsch A (2010) BISMA–fast and accurate bisulfite sequencing data analysis of individual clones from unique and repetitive sequences. BMC Bioinformatics 11: 230. 20459626 10.1186/1471-2105-11-230 PMC2877691

[32] Watanabe D, Suetake I, Tada T, Tajima S (2002) Stage- and cell-specific expression of Dnmt3a and Dnmt3b during embryogenesis. Mech Dev 118: 187–190. 12351185 10.1016/s0925-4773(02)00242-3

[33] Feng J, Chang H, Li E, Fan G (2005) Dynamic expression of de novo DNA methyltransferases Dnmt3a and Dnmt3b in the central nervous system. J Neurosci Res 79: 734–746. 15672446 10.1002/jnr.20404

[34] Singh RP, Shiue K, Schomberg D, Zhou FC (2009) Cellular epigenetic modifications of neural stem cell differentiation. Cell Transplant 18: 1197–1211. 19660178 10.3727/096368909X12483162197204 PMC2812652

[35] Sen GL, Reuter JA, Webster DE, Zhu L, Khavari PA (2010) DNMT1 maintains progenitor function in self-renewing somatic tissue. Nature 463: 563–567. 20081831 10.1038/nature08683 PMC3050546

[36] Liu Y, Sun L, Jost JP (1996) In differentiating mouse myoblasts DNA methyltransferase is posttranscriptionally and posttranslationally regulated. Nucleic Acids Res 24: 2718–2722. 8759002 10.1093/nar/24.14.2718 PMC145988

[37] D’Aiuto L, Di Maio R, Mohan KN, Minervini C, Saporiti F, et al (2011) Mouse ES cells overexpressing DNMT1 produce abnormal neurons with upregulated NMDA/NR1 subunit. Differentiation 82: 9–17. 21492995 10.1016/j.diff.2011.03.003 PMC3115397

[38] Chen T, Ueda Y, Dodge JE, Wang Z, Li E (2003) Establishment and maintenance of genomic methylation patterns in mouse embryonic stem cells by Dnmt3a and Dnmt3b. Mol Cell Biol 23: 5594–5605. 12897133 10.1128/MCB.23.16.5594-5605.2003 PMC166327

[39] Rountree MR, Bachman KE, Baylin SB (2000) DNMT1 binds HDAC2 and a new co-repressor, DMAP1, to form a complex at replication foci. Nat Genet 25: 269–277. 10888872 10.1038/77023

[40] Fuks F, Hurd PJ, Wolf D, Nan X, Bird AP, et al (2003) The methyl-CpG-binding protein MeCP2 links DNA methylation to histone methylation. J Biol Chem 278: 4035–4040. 12427740 10.1074/jbc.M210256200

[41] Bachman KE, Rountree MR, Baylin SB (2001) Dnmt3a and Dnmt3b are transcriptional repressors that exhibit unique localization properties to heterochromatin. J Biol Chem 276: 32282–32287. 11427539 10.1074/jbc.M104661200

[42] Deplus R, Brenner C, Burgers WA, Putmans P, Kouzarides T, et al (2002) Dnmt3L is a transcriptional repressor that recruits histone deacetylase. Nucleic Acids Res 30: 3831–3838. 12202768 10.1093/nar/gkf509 PMC137431

[43] Schroeder DI, Lott P, Korf I, LaSalle JM (2011) Large-scale methylation domains mark a functional subset of neuronally expressed genes. Genome Res 21: 1583–1591. 21784875 10.1101/gr.119131.110 PMC3202276

[44] Chen T, Tsujimoto N, Li E (2004) The PWWP domain of Dnmt3a and Dnmt3b is required for directing DNA methylation to the major satellite repeats at pericentric heterochromatin. Mol Cell Biol 24: 9048–9058. 15456878 10.1128/MCB.24.20.9048-9058.2004 PMC517890

[45] Ge YZ, Pu MT, Gowher H, Wu HP, Ding JP, et al (2004) Chromatin targeting of de novo DNA methyltransferases by the PWWP domain. J Biol Chem 279: 25447–25454. 14998998 10.1074/jbc.M312296200

[46] Li JY, Pu MT, Hirasawa R, Li BZ, Huang YN, et al (2007) Synergistic function of DNA methyltransferases Dnmt3a and Dnmt3b in the methylation of Oct4 and Nanog. Mol Cell Biol 27: 8748–8759. 17938196 10.1128/MCB.01380-07 PMC2169413

[47] Jessell TM (2000) Neuronal specification in the spinal cord: inductive signals and transcriptional codes. Nat Rev Genet 1: 20–29. 11262869 10.1038/35049541

[48] Stremenova J, Krepela E, Mares V, Trim J, Dbaly V, et al (2007) Expression and enzymatic activity of dipeptidyl peptidase-IV in human astrocytic tumours are associated with tumour grade. Int J Oncol 31: 785–792. 17786309

[49] Sedo A, Krepela E, Kasafirek E, Kraml J, Kadlecova L (1991) Dipeptidyl peptidase IV in the human lung and spinocellular lung cancer. Physiol Res 40: 359–362. 1684289

[50] Du J, Fan Z, Ma X, Gao Y, Wu Y, et al (2011) Expression of Dpp6 in mouse embryonic craniofacial development. Acta Histochem 113: 636–639. 20817268 10.1016/j.acthis.2010.08.001

