# Supplementary figures and images for: Epigenetic Regulation of Dpp6 Expression by Dnmt3b and Its Novel Role in the Inhibition of RA Induced Neuronal Differentiation of P19 Cells
Source: PLoS One. 2013 Feb 7;8(2):e55826. doi: 10.1371/journal.pone.0055826 (PMC3567024; doi:10.1371/journal.pone.0055826)

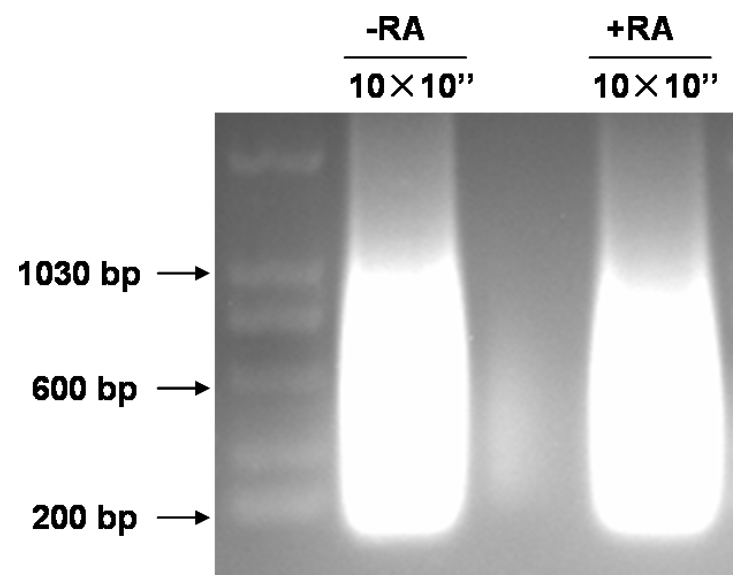


**Figure S1**

Supplement: Figure S1 — P19 cells either untreated or RA treated for initial 2 days and further cultured for 4 days without RA were formaldehyde fixed, lysed, and sonicated for ten 10 sec pulses (10 sec on, 10 sec off) to yield DNA fragments with an average size of ∼500 bp. (DOC) [file pone.0055826.s001.doc]

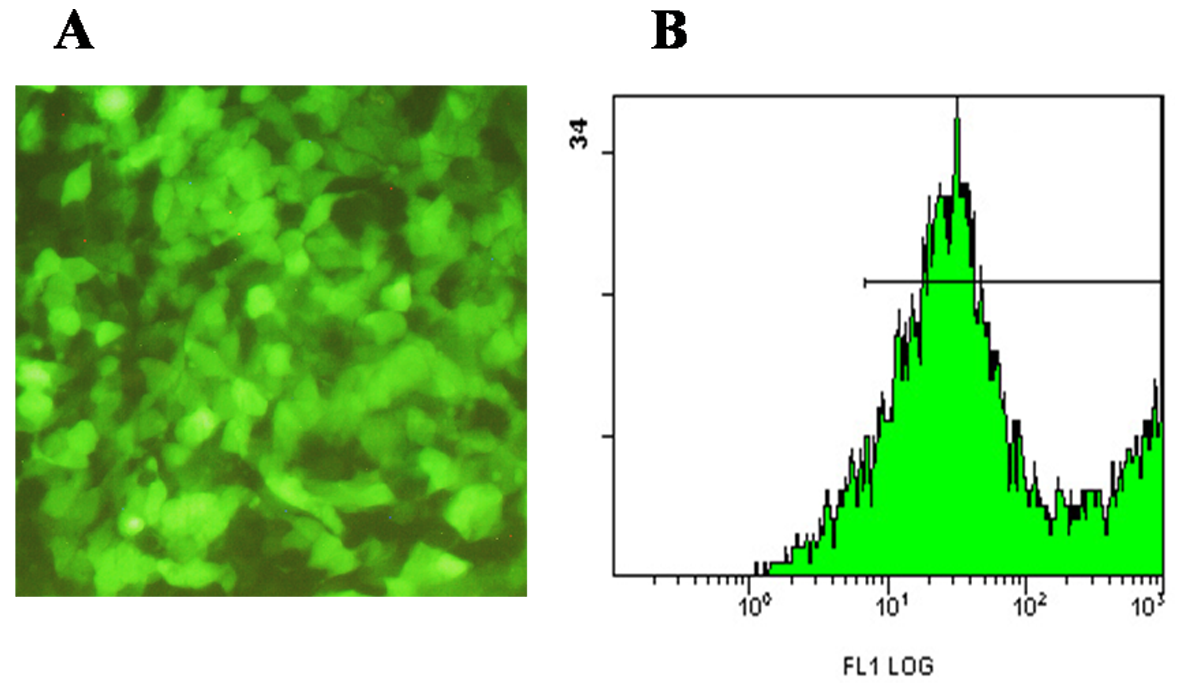


**Figure S2**

Supplement: Figure S2 — A&B, P19 cells were infected with lentiviral particles that contain GFP as marker to monitor infection efficiency. At optimal dose, more than 90% cells were GFP positive as determined by flow cytometry. (DOC) [file pone.0055826.s002.doc]

**Table S1**


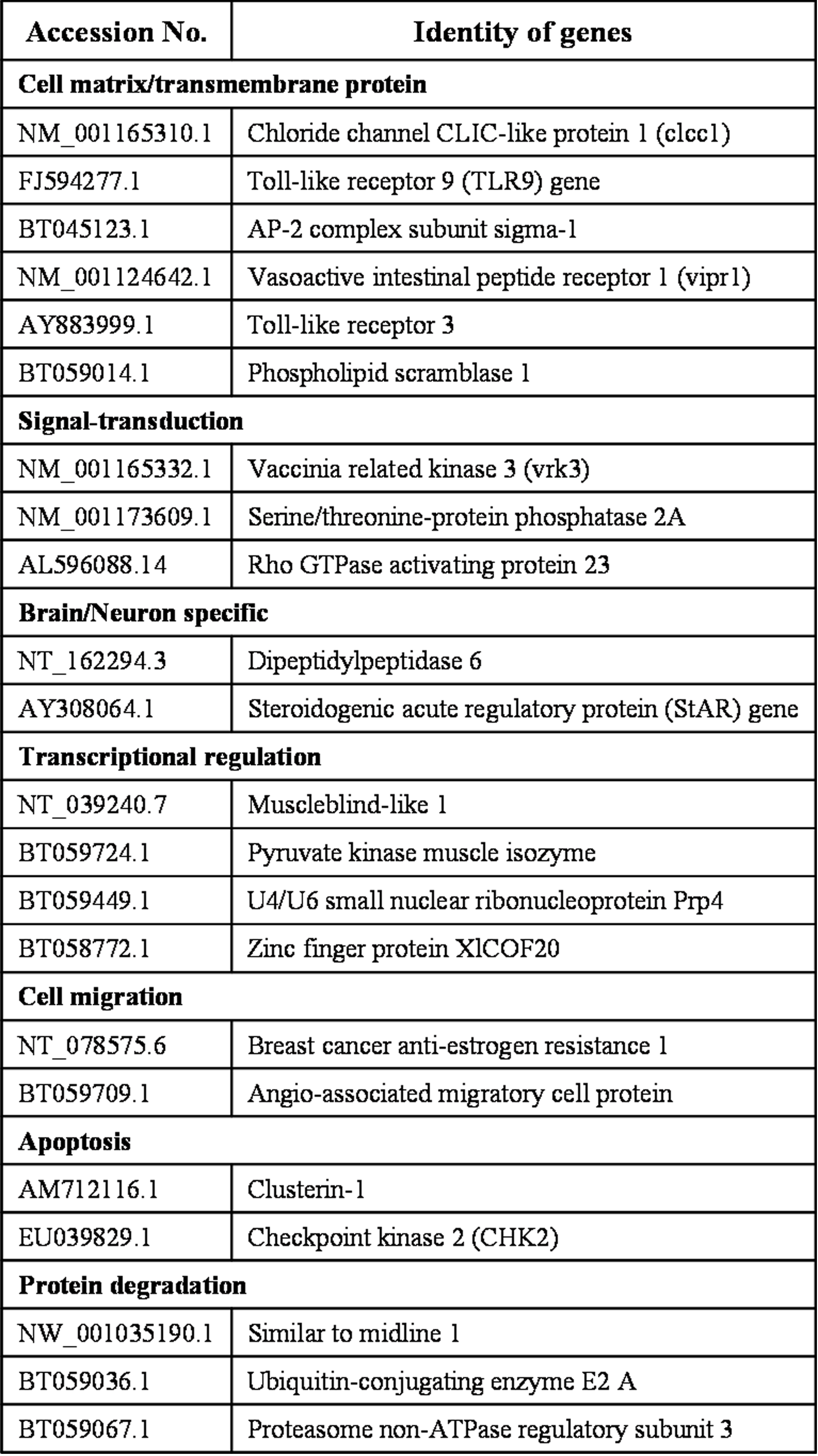

Supplement: Table S1 — Dnmt3b target genes in P19 derived neurons. (DOC) [file pone.0055826.s003.doc]
